# Supplementary material for: Correcting the hebbian mistake: Toward a fully error-driven hippocampus
Source: PLoS Comput Biol. 2022 Oct 11;18(10):e1010589. doi: 10.1371/journal.pcbi.1010589 (PMC9586412; doi:10.1371/journal.pcbi.1010589)
Supplement: S1 Appendix — (PDF) [file pcbi.1010589.s001.pdf]

In this appendix we provide some of the key parameters to understanding how the Theremin model is structured, and organized to do the AB-AC memory task. See table captions for detailed descriptions on parameters/diagrams. The best documentation for those interested in all the details is the fully-functioning Theremin model along with further detailed documentation, available at: `https://github.com/ccnlab/hip-edl`.

S1 Table shows the specific layer sizes associated with the small, medium, and large networks, and S1 Fig shows an example of training and testing patterns representing pools of EC layer activations.

1 Implementational Details

The model was implemented using the Leabra framework, which is described in detail in these sources: `https://github.com/emer/leabra` [1], [2], and summarized here. These same equations and default parameters have been used to simulate over 40 different models in [1] and [2], and a number of other research models. Thus, the model can be viewed as an instantiation of a systematic modeling framework using standardized mechanisms, instead of constructing new mechanisms for each model.

The basic activation dynamics are based on standard electrophysiological principles of real neurons, as captured by the AdEx (adapting exponential) model of Gerstner and colleagues [3], using a rate code approximation that produces a graded activation signal matching the actual instantaneous rate of spiking across a population of AdEx neurons. We generally conceive of a single rate-code neuron as representing a microcolumn of roughly 100 spiking pyramidal neurons in the neocortex.

The excitatory synaptic input conductances (i.e., net input) is computed as an average, not a sum, over connections, based on normalized, sigmoidaly transformed weight values, which are subject to scaling on a projection level to alter relative contributions. Automatic scaling is performed to compensate for differences in expected activity level in the different projections.

Inhibition is computed using a feed-forward (FF) and feed-back (FB) inhibition function (FFFB) that closely approximates the behavior of inhibitory interneurons in the neocortex. FF is based on a multiplicative factor applied to the average net input coming into a layer, and FB is based on a multiplicative factor applied to the average activation within the layer. These simple linear functions do an excellent job of controlling the overall activation levels in bidirectionally connected networks, producing behavior very similar to the more abstract computational implementation of kWTA dynamics implemented in previous versions.

There is a single learning equation, derived from a detailed model of spike timing dependent plasticity (STDP) by [4], that produces a combination of Hebbian associative and error-driven learning. For historical reasons, we call this the XCAL equation (eXtended Contrastive Attractor Learning), and it is functionally very similar to the BCM learning rule developed by [5]. The essential learning dynamic involves a Hebbian co-product of sending neuron activation times receiving neuron activation, which biologically reflects the amount of calcium entering through NMDA channels, and this co-product is then compared against a floating threshold value. To produce the Hebbian learning dynamic, this floating threshold is based on a long-term running average of the receiving neuron activation. This is the key idea for the BCM algorithm. To produce error-driven learning, the floating threshold is based on a much faster running average of activation co-products, which reflects an expectation or prediction, against which the instantaneous, later outcome is compared.

Weights are subject to a contrast enhancement function, which compensates for the soft (exponential) weight bounding that keeps weights within the normalized 0-1 range. Contrast enhancement is important for enhancing the selectivity of self-organizing

learning, and generally results in faster learning with better overall results. Learning operates on the underlying internal linear weight value. Biologically, we associate the underlying linear weight value with internal synaptic factors such as actin scaffolding, CaMKII phosphorlation level, etc, while the contrast enhancement operates at the level of AMPA receptor expression.

The following shows the main equations used to simulate neural activity and learning (see `https://github.com/emer/leabra` in the README.md for complete details and discussion).

1.1 Activation Equations

- `GeRaw += Sum_(recv) Prjn.GScale * Send.Act * Wt`
  - `Prjn.GScale` is the Input Scaling factor that includes 1/N to compute an average, and the `WtScaleParams Abs` absolute scaling and `Rel` relative scaling, which allow one to easily modulate the overall strength of different input projections.
- `Ge += (1/ DtParams.GTau) * (GeRaw - Ge)`
  - This does a time integration of excitatory conductance, `GTau = 1.4` default for 1 msec default cycle.
- `ffi = FFFBParams.FF * MAX(avgGe - FFBParams.FF0, 0)`
  - feedforward component of inhibition with FF multiplier (1 by default) -- has FF0 offset and can't be negative (that's what the MAX(.. ,0) part does).
  - `avgGe` is average of Ge variable across relevant Pool of neurons, depending on what level this is being computed at, and `maxGe` is max of Ge across Pool
- `fbi += (1 / FFFBParams.FBTau) * (FFFBParams.FB * avgAct - fbi)`
  - feedback component of inhibition with FB multiplier (1 by default) -- requires time integration to dampen oscillations that otherwise occur -- FBTau = 1.4 default.
- `Gi = FFFBParams.Gi * (ffi + fbi)`
  - total inhibitory conductance, with global Gi multiplier -- default of 1.8 typically produces good sparse distributed representations in reasonably large layers (25 units or more).
- `geThr = (Gi * (Erev.I - Thr) + Gbar.L * (Erev.L - Thr) / (Thr - Erev.E)`
- `nwAct = NoisyXX1(Ge * Gbar.E - geThr)`
  - geThr = amount of excitatory conductance required to put the neuron exactly at the firing threshold, `XX1Params.Thr` = .5 default, and NoisyXX1 is the x / (x+1) function convolved with gaussian noise kernel, where x = `XX1Parms.Gain * (Ge - geThr)` and Gain is 100 by default
- `Act += (1 / DTParams.VmTau) * (nwAct - Act)`
  - time-integration of the activation, using same time constant as Vm integration (VmTau = 3.3 default)

- `Vm += (1 / DTParams.VmTau) * Inet`
- `Inet = Ge * (Erev.E - Vm) + Gbar.L * (Erev.L - Vm) + Gi * (Erev.I - Vm) + Noise`
  - Membrane potential computed from net current via standard RC model of membrane potential integration. In practice we use normalized Erev reversal potentials and Gbar max conductances, derived from biophysical values: Erev.E = 1, .L = 0.3, .I = 0.25, Gbar's are all 1 except Gbar.L = .2 default.

1.2 Learning Equations

- `AvgSS += (1 / SSTau) * (Act - AvgSS)`
  - super-short time scale running average, SSTau = 2 default, which is first pass of sequence of running-average integrations of activity that drive temporal-difference learning.
- `AvgS += (1 / STau) * (AvgSS - AvgS)`
  - short time scale running average, STau = 2 default -- this represents the *plus phase* or actual outcome signal in comparison to AvgM
- `AvgM += (1 / MTau) * (AvgS - AvgM)`
  - medium time-scale running average, MTau = 10 -- this represents the *minus phase* or expectation signal in comparison to AvgS
- `AvgL += (1 / Tau) * (Gain * AvgM - AvgL); AvgL = MAX(AvgL, Min)`
  - long-term running average -- this is computed just once per learning trial, *not every cycle* like the ones above -- params on `AvgLParams`: Tau = 10, Gain = 2.5 (this is a key param -- best value can be lower or higher) Min = .2
- `AvgLLrn = ((Max - Min) / (Gain - Min)) * (AvgL - Min)`
  - learning strength factor for how much to learn based on AvgL floating threshold -- this is dynamically modulated by strength of AvgL itself, and this turns out to be critical -- the amount of this learning increases as units are more consistently active all the time (i.e., "hog" units). Params on `AvgLParams`, Min = 0.0001, Max = 0.5. Note that this depends on having a clear max to AvgL, which is an advantage of the exponential running-average form above.
- `AvgLLrn *= MAX(1 - layCosDiffAvg, ModMin)`
  - also modulate by time-averaged cosine (normalized dot product) between minus and plus phase activation states in given receiving layer (layCosDiffAvg), (time constant 100) -- if error signals are small in a given layer, then Hebbian learning should also be relatively weak so that it doesn't overpower it -- and conversely, layers with higher levels of error signals can handle (and benefit from) more Hebbian learning. The MAX(ModMin) (ModMin = .01) factor ensures that there is a minimum level of .01 Hebbian (multiplying the previously-computed factor above). The .01 * .05 factors give an upper-level value of .0005 to use for a fixed constant AvgLLrn value -- just slightly less than this (.0004) seems to work best if not using these adaptive factors.

- `AvgSLrn = (1-LrnM) * AvgS + LrnM * AvgM`
  - mix in some of the medium-term factor into the short-term factor -- this is important for ensuring that when neuron turns off in the plus phase (short term), that enough trace of earlier minus-phase activation remains to drive it into the LTD weight decrease region -- LrnM = .1 default.
- `srs = Send.AvgSLrn * Recv.AvgSLrn`
- `srm = Send.AvgM * Recv.AvgM`
- `dwt = XCAL(srs, srm) + Recv.AvgLLrn * XCAL(srs, Recv.AvgL)`
  - weight change is sum of two factors: error-driven based on medium-term threshold (srm), and BCM Hebbian based on long-term threshold of the recv unit (Recv.AvgL)
- XCAL is the "check mark" linearized BCM-style learning function that was derived from the Urakubo Et Al (2008) STDP model, as described in more detail in the CCN Textbook: `https://CompCogNeuro.org`
  - `XCAL(x, th) = (x < DThr) ? 0 : (x > th * DRev) ? (x - th) : (-x * ((1-DRev)/DRev))`
  - DThr = 0.0001, DRev = 0.1 defaults, and x ? y : z terminology is C syntax for: if x is true, then y, else z
- `DWt *= (DWt > 0) ? Wb.Inc * (1-LWt) : Wb.Dec * LWt`
  - `LWt` is the linear, non-contrast enhanced version of the weight value, and `Wt` is the sigmoidal contrast-enhanced version, which is used for sending netinput to other neurons. One can compute LWt from Wt and vice-versa, but numerical errors can accumulate in going back-and forth more than necessary, and it is generally faster to just store these two weight values.
  - soft weight bounding -- weight increases exponentially decelerate toward upper bound of 1, and decreases toward lower bound of 0, based on linear, non-contrast enhanced LWt weights. The `Wb` factors are how the weight balance term shift the overall magnitude of weight increases and decreases.
- `LWt += DWt`
  - increment the linear weights with the bounded DWt term
- `Wt = SIG(LWt)`
  - new weight value is sigmoidal contrast enhanced version of linear weight
  - `SIG(w) = 1 / (1 + (Off * (1-w)/w)^Gain)`
- `DWt = 0`
  - reset weight changes now that they have been applied.

References

1. O'Reilly RC, Munakata Y, Frank MJ, Hazy TE, Contributors. Computational Cognitive Neuroscience. Wiki Book, 1st Edition, URL: http://ccnbook.colorado.edu; 2012. Available from: `http://ccnbook.colorado.edu`.

2. O'Reilly RC, Munakata Y. Computational Explorations in Cognitive Neuroscience: Understanding the Mind by Simulating the Brain. Cambridge, MA: MIT Press; 2000.

3. Brette R, Gerstner W. Adaptive Exponential Integrate-and-Fire Model as an Effective Description of Neuronal Activity. Journal of Neurophysiology. 2005;94(5):3637–3642. doi:10.1152/jn.00686.2005.

4. Urakubo H, Honda M, Froemke RC, Kuroda S. Requirement of an Allosteric Kinetics of NMDA Receptors for Spike Timing-Dependent Plasticity. The Journal of Neuroscience. 2008;28(13):3310–3323.

5. Bienenstock EL, Cooper LN, Munro PW. Theory for the Development of Neuron Selectivity: Orientation Specificity and Binocular Interaction in Visual Cortex. The Journal of Neuroscience. 1982;2(2):32–48.
