# Supplementary material for: Correcting the hebbian mistake: Toward a fully error-driven hippocampus
Source: PLoS Comput Biol. 2022 Oct 11;18(10):e1010589. doi: 10.1371/journal.pcbi.1010589 (PMC9586412; doi:10.1371/journal.pcbi.1010589)
Supplement: S1 Table — In neural networks, larger network size usually leads to higher capacity, when controlled for other settings. In the current study, we tested different variations of the hippocampus model for three different network sizes to show the benefit of error-driven learning for hippocampus regardless of sizes, meaning the mechanism is generalizable. For pool sizes, the numbers in the table refer to number of neurons in that specific pool. Note: DG size is around five times CA3 size as specified in our previous model [21]. (PDF) [file pcbi.1010589.s003.pdf]

Parameter \ Network Size	Small	Medium	Large
Input Pool Size	7x7	7x7	7x7
Input Number of Pools	2x3	2x3	2x3
ECin Pool Size	7x7	7x7	7x7
ECin Number of Pools	2x3	2x3	2x3
ECout Pool Size	7x7	7x7	7x7
ECout Number of Pools	2x3	2x3	2x3
DG Size	44x44	67x67	89x89
CA3 Size	20x20	30x30	40x40
CA1 Pool Size	10x10	15x15	20x20
CA1 Number of Pools	2x3	2x3	2x3

S1 Table. Parameters for network sizes. In neural networks, larger network size usually leads to higher capacity, when controlled for other settings. In the current study, we tested different variations of the hippocampus model for three different network sizes to show the benefit of error-driven learning for hippocampus regardless of sizes, meaning the mechanism is generalizable. For pool sizes, the numbers in the table refer to number of neurons in that specific pool. Note: DG size is around five times CA3 size as specified in our previous model [1].

References

1. Ketz N, Morkonda SG, O'Reilly RC. Theta Coordinated Error-Driven Learning in the Hippocampus. PLoS Computational Biology. 2013;9:e1003067.
