# Supplementary material for: Correcting the hebbian mistake: Toward a fully error-driven hippocampus
Source: PLoS Comput Biol. 2022 Oct 11;18(10):e1010589. doi: 10.1371/journal.pcbi.1010589 (PMC9586412; doi:10.1371/journal.pcbi.1010589)
Supplement: S2 Table — Non-default parameters used in the model, with default shown. (PDF) [file pcbi.1010589.s004.pdf]

Area	Param	Value	Default
ECin	Inhib.Pool.Gi	2	1.8
ECout	Inhib.Pool.Gi	2	1.8
ECout	CA1ToECout.WtScale.Abs	4	1
CA1	Inhib.Pool.Gi	2.4	1.8
CA1	CA3ToCA1.CHL.Hebb	0.01	0.001
DG	Inhib.Layer.Gi	3.8	1.8
DG	ECinToDG.CHL.Hebb	0.2	0.001
DG	ECinToDG.CHL.SAvgCor	0.1	0.4
CA3	Inhib.Layer.Gi	2.8	1.8
CA3	DGToCA3.CHL.Hebb	0.01	0.001

S2 Table. Parameters. Non-default parameters used in the model, with default shown.
