# Supplementary material for: Correcting the hebbian mistake: Toward a fully error-driven hippocampus
Source: PLoS Comput Biol. 2022 Oct 11;18(10):e1010589. doi: 10.1371/journal.pcbi.1010589 (PMC9586412; doi:10.1371/journal.pcbi.1010589)
Supplement: Attachment — Submitted filename: Review.docx [file pcbi.1010589.s005.docx]

**Review (PCOMPBIOL-D-21-02014): Zheng, Liu, Nishiyama, Ranganath, and O'Reilly**

Zheng et al. present a solid and well-written modeling study that follows up on a previous paper from this group (Ketz, 2013). This paper borrows the same hippocampal architecture but examines variations of the learning rules at critical perforant path and mossy fiber projections to CA3 synapses. Their findings demonstrate the benefits to memory capacity, training performance, and (arguably) biological fidelity of a certain class of error-driven learning (EDL) rules. Recent advances in AI and theoretical neuroscience have increasingly focused on the importance of predictions and error signals for learning, inference, and planning. This paper provides considerable exposition to motivate critical questions about error signals and error-driven plasticity in the hippocampus with respect to its role in episodic memory formation and retrieval. A key principle of episodic memory is that maximizing future retrieval requires minimizing potential representational interference between events with similar sensory and spatiotemporal properties. Thus, we need to better understand the dynamical interplay between pattern separation processes and the pattern completion that drives CA3-based autoassociative retrieval. This paper makes a clear contribution toward this understanding and should be of major interest to the field. My main concerns involve the authors' framing of Hebbian learning as a baseline for their results and whether the current set of modeling results as presented are sufficient to substantiate the broader claims. It was an enjoyable read and is appropriate for the journal; however, the presentation and results should be strengthened to both better connect with the literature and provide additional context for the audience.

*Major comments:*

(1) The abstract and other places state the main problem with Hebbian learning as weight modification that continues "unnecessarily beyond" what is sufficient for retrieval. In some places, the authors refer to this naïve notion as the "simple Hebbian approach" (e.g., start of intro, para 4). While a simplistic ∆w=µxy rule encapsulates the old "fire together, wire together" dictum, I believe it is widely understood that such a learning rule is impractical, given its tendency to explode. That is, the foil in the authors' story is a straw man. The authors might have in mind some self-normalizing variant of generalized Hebbian plasticity (e.g., Oja's rule, BCM, etc.), but they don't mention it directly or discuss comparisons with other Hebb-like variations. The main issue with this is that implying "simple Hebb" to be an appropriate baseline for comparison is both unfair to Hebb and overgenerous to the authors' evaluation of their EDL models' performance. The paper's impact, and its connection to the comp/theory literature since the Ketz (2013) paper, would substantively benefit from clarifying throughout the manuscript precisely what is meant by "Hebbian learning", both in general discussion and when describing implementations in the 2013 model and this model (e.g., the ECin → DG pathway). Since the gap being studied here is Hebbian → EDL, any obscurity around "Hebbian" terminology detracts from the interpretability of the results.
(2) Relatedly, the scientific logic motivating the study of RW-style EDL rules established in the introduction (para 4) is rather weak. That logic could be paraphrased: "To avoid simple Hebb we need self-limited learning; 'one well-established class' is EDL; therefore we incorporate EDL and show that it outperforms simple Hebb." This logic undercuts itself at 'one well-established class', both because it's an appeal to authority and it hints at other classes which the authors fail to mention or study. This latter point raises questions (in a reasonable reader) of why this particular formulation of EDL was chosen for study from among the wider universe of self-limiting learning rules, whether well-established or not. Its relationship to error backpropagation is potentially interesting, but it doesn't substantiate its support in biology (which is fine, it's just that there are presumably many other self-limiting rules with varying levels of biological evidence and plausibility). If this paper is pushing us toward an "error-driven hippocampus", then what we (the field) need to see is the comparison between EDL rules and some spanning set of non-EDL self-limiting rules. (I might be mistaken, but I think the authors' NoEDL variant here simply disabled CA3 synaptic modification and the ThetaPhase variant utilized "Hebbian learning"; there is no test of non-EDL self-limited learning at those synapses.) If self-limited learning is the logical alternative to the Hebbian "mistake", then why isn't self-limited learning studied and explicated more broadly (within the context of this hippocampal architecture/model)? If EDL emerges as the self-limiting "winner" (on grounds of capacity, performance, or biological fidelity), then that makes a much stronger case. To fully support the authors' claims, I would suggest that they expand their investigation, to a reasonable extent, to provide some baseline of comparison with non-EDL self-limiting rules (e.g., FORCE learning, synaptic resource allocation, etc.).

*Minor points:*

(3) The paper's title is perhaps too strongly worded, since "Hebbian Mistake" implicates Hebb in an error and conjures up something of a straw man (cf.  comments 1&2 above). I can only suggest (sincerely suggest; I'm not demanding a title change here) that the authors reconsider whether the title strikes the right note given the study's claims.
(4) Figure 1: Please clarify the relationship between the 4 "quarters" and the theta cycle. It would be helpful to have a simple diagram showing the correspondence of Q1-Q4 aligned with a sinusoidal theta wave. Also, it looks like Q2 and Q3 are identical? Do those quarters correspond to the cycle 25-75 interval (with the big-loop signals) in Figure 6?
(5) Following on comment #3: It might be helpful to add a brief overview of the "nuts and bolts" of the model implementation (perhaps in the Methods section) that the authors could reference at this point, since many model details seem to be glossed over or presumed to be found in the 2013 paper. This should include at least brief descriptions of the units, activations, and layers an explanation of simulation timing relating time steps to cycles to quarters to epochs to train/test trials. These relationships won't be immediately obvious to most readers, and it shouldn't be necessary to go to the 2013 paper for basic information.
(6) p.5, following Eq (4): "ECin is _the_ sending activity into CA3"; I'm assuming that 'the' is a typo?
(7) p.7, para 1: "Figure 1 shows the _standard_ hippocampal architecture...". The word 'standard' shouldn't really be used to describe these models. This architecture might be standard in the authors' labs, but there are many ways to construct models. There are other instances of "standard" in the manuscript which should be similarly struck.
(8) While "NEpochs" and "ABmem" work fine as variable names, they are awkward to read and difficult to remember. The authors could just as well use "N" and "C" (or a similarly readable refactoring) to refer to training epochs and memory capacity.
(9) Are the Theremin and ThetaPhase curves shown in Figure 4 the same as those in Figure 3? If so, that should be clarified, preferably in one or both captions. Or, the authors could reconsider the presentation of this data to prevent reader confusion.
(10) Figure 5: The text does mention the difference in training epochs between Theremin (10) and NoEDL (30), but these plots with differently scaled x-axes in the left and right columns are not intuitively interpretable. Because the epochs are discrete, having fewer in the Theremin plots means that the visual slope of the connecting lines has a different meaning regarding the learning dynamics of the model w.r.t. the NoEDL plots. Given the relative complexity of the data (esp. the similarity curves), the ease of interpretation of this figure would greatly benefit from an attempt to horizontally stretch (half of) the plots so that the x-axis scales are preserved over all of them.
(11) Figure 6: It would be very helpful to add some dotted lines or arrows to indicate the cycles at which DG input turns on (25). Also, something clearly happens around cycle 75 at the end of Q3, but the text and caption don't make this very clear. This could be clarified in the Methods as I suggested above or in other ways as the authors see fit.
(12) Discussion, p.15, para 3 "Another class...": This paragraph discusses the long-running class of sequence learning models, but it doesn't come to a very satisfying conclusion. How exactly are we supposed to interpret the current study's results in the context of sequence learning, which depends precisely on the kinds of learned representational overlaps that the authors' CA3 EDL rule and EC → DG plasticity work to remove? The authors' seem to dismiss this entire literature by stating that "most" of these models synthesize predictions. If it's only most that can be thusly dismissed, then the ones that remain need to be addressed, no? How would Chip Levy's local context units fare in an EDL regime?
(13) Discussion, Novel Predictions, second bullet point: Since the promise of experimental predictions was prominent in the abstract and other places, it's not clear why the authors did not already undertake to investigate some of these pathway-specific modulations in their model. As written, the predictions are very vague: "should affect error-driven learning", "the specific temporal dynamics associated...". Given the effort and expense of experimental corroboration, it would be more impactful to more clearly and precisely demonstrate hypothesis-driven results from pathway lesion/modulation interventions in the Theremin model to both inspire and guide experimentalists who may be interested in these questions.
