# Supplementary material for: Correcting the hebbian mistake: Toward a fully error-driven hippocampus
Source: PLoS Comput Biol. 2022 Oct 11;18(10):e1010589. doi: 10.1371/journal.pcbi.1010589 (PMC9586412; doi:10.1371/journal.pcbi.1010589)
Supplement: Attachment — Submitted filename: Zheng et al 2022 Review.docx [file pcbi.1010589.s006.docx]

The manuscript submitted by Zheng et al. details a compelling model in which synaptic strengths of connections in the medial temporal lobe are updated during specific phases of a theta cycle according to error-driven learning rules. The model focuses on key regions of the medial temporal lobe in which hippocampal synaptic weights can be adjusted (CA1 inputs to EC, EC inputs to CA3, or CA3 recurrent collateral inputs) to yield activity in CA1 or CA3 that matches target activity patterns (driven by EC or DG, respectively). The authors note that such error-driven learning rules provide the advantage of limiting synaptic weight changes to only those necessary to faithfully represent input patterns, mitigating interference created by excessive weight changes and increasing overall memory capacity. I have several broad questions regarding the goal of achieving specific target activity patterns in this system and the necessity of the theta-constrained timing to achieve the temporal differences in activation state. I also have a number of recommendations that I believe will improve the clarity and impact of this work.

Major Comments:

1. One of the central goals of this model (and a previous model published by this group) is to adjust synaptic weights over the course of learning until EC­_OUT_ matches EC_IN_, with the specific addition of adjustments in this model until CA3 _DG_ − CA3 _OUT_. I am familiar with the history of the CLS model and the advantage that the new adjustments in this model provide for reducing interference. The adjustments in the original version of this model (EC­_OUT_ matches EC_IN_) require a bit more explanation. What are the goals/advantages of having EC­_OUT_ match that of EC_IN_? This seems to imply that CA1 is being optimized to provide faithful representations of original EC input, rather than performing any unique transformations. The *dW* = CA1(EC­_OUT_ - EC_IN_) seems to negate somewhat the advantages afforded by *dW* = EC(CA3­_DG_ – CA3_NODGE_). In other words, the structure of *dW* = CA1(EC­_OUT_ - EC_IN_) seems like it disrupts the ability of CA1 to output unique separations, associations, or other transformations formed *within* the hippocampus. Moreover, it makes it challenging to incorporate strong recent evidence that entorhinal activity is heavily influenced by hippocampal activity. It might be interesting to probe CA1 activity patterns of EC _OUT_ without *dW* = CA1(EC­_OUT_ - EC_IN_) while still retaining *dW* = EC(CA3­_DG_ – CA3_NODGE_) to provide insight into how *dW* = CA1(EC­_OUT_ - EC_IN_) influences hippocampal computations/interference (and then change the name of the noEDL condition).
2. The authors attempt to leverage the unique timing of different inputs within a theta cycle in order to allow different patterns of activity to manifest across a region at distinct times. While I think it is a great idea to consider a major temporal organizing principle of the region, I am not sure the model needs to be nested within a theta timescale. Attempting to do so makes it fragile to scrutiny of the feasibility of the timescale and potentially less applicable to primate models where the theta rhythm is less prominent. The authors include some citations indicating that plasticity within a theta timescale may work (e.g. Hasselmo et al. 2002), but the work was targeting projections to CA1, which are not the target of their error-driven learning models. Synaptic plasticity of other synapses may obey very different temporal rules, and a more recent study they cite from Quirk et al. suggests that rhythms facilitating learning can be quite broad (i.e. <10Hz). Synaptic plasticity of other synapses may obey very different temporal rules. I think that the authors can maintain the theta cycle structure of the current model, but perhaps provide context that it is a valuable framework here for achieving their goals rather than *the* only hypothesized mechanism for error-driven learning. They can provide context in the introduction and discussion with any additional references justifying their timing and other possible timing mechanisms.
3. The authors do a great job explaining the relevant connectivity. However, there are occasions where it is not clear which connections are undergoing Hebbian learning and which connections are undergoing error-driven learning. For example, there are places in the text where the language can read as if there is EDL at CA3 inputs to CA1. Figure 1 is extremely helpful in detailing the connections that are giving rise to expected patterns of activity at different phases of the theta cycle. Perhaps this figure can be adapted to indicate (perhaps through dotted or dashed lines versus solid lines) which connections have EDL, which ones have Hebbian learning, and which (if any) have no plasticity. In addition, perhaps patterns of activity can be labeled as CA3_DG_ (or “target”) versus CA3_NODGE_ (or “expected”) when they occur in the upper panels, and similarly for other patterns utilized for EDL. Adding the equations next to the connections undergoing EDL would also be tremendously helpful.

Minor Comments

1. The acronym Theremin, while fun, is an odd combination of letters from the model name, has an extra E, and perhaps doesn’t capture the description or goals of the model well. I understand that model names are often somewhat personal, but I think that a different name would increase the impact and accessibility long-term. In addition, as there are only two synapses with EDL, I suggest removing the word Total from the model name.
2. I find it hard to interpret the tables at the end of the manuscript, particularly the testing table. I am not exactly sure what is to be learned from these table. If no input is given for the B/C pool, then why do several pools have Context B#/C# labels? In addition, B#/C# goes to 4, when this notation is used in the test to indicate a single pair of a # of possible pairs much greater than 4. Perhaps the two tables can be combined into a figure detailing how the inputs to the a subset or all pools evolve across training and test.
3. The authors experiment with plasticity at EC-DG synapses, and their results suggest that favoring LTD increases separation/reduces interference. Perhaps the authors could relate this finding to what is known about homeostatic synaptic plasticity at these synapses (Hsu et al., *The dentate gyrus as a filter or gate: a look back and a look ahead*, 2007)
4. The authors relate there model to several other models and comment on the ability of the hippocampus to generate predictions. It would be interesting if they could also relate their model to Jung et al. 2018 (*Remembering rewarding futures: A simulation‐selection model of the hippocampus*).
5. There is a typo in the Figure 2A description “traind.”
