# Supplementary material for: Correcting the hebbian mistake: Toward a fully error-driven hippocampus
Source: PLoS Comput Biol. 2022 Oct 11;18(10):e1010589. doi: 10.1371/journal.pcbi.1010589 (PMC9586412; doi:10.1371/journal.pcbi.1010589)
Supplement: Attachment — Submitted filename: responses_sub2.docx [file pcbi.1010589.s007.docx]

Here we describe our responses to the paper. We have extensively revised the paper, and have included a "diff" version that tracks the changes so you can easily see what was changed. We feel that the paper is much improved as a result of the reviewer input, and are hopeful that the reviewers and editor agree.

# Reviewer #1:

1. My main concern is that the authors assume the reader will know or read a whole series of prior papers presenting models upon which this one is built – the methods presented here are simply inadequate for a reader to fully understand, yet alone reproduce the work. The authors send the reader to a githib repository, but even there basic features are unclear – for example, whether “Adaptive Exponential Leaky Integrate and Fire” model neurons are used, or firing-rate models based on the latter. These are the basic building blocks of any model, along with the impacts of connections – amplitudes and time constants, most importantly. The reader should not have to wade through all the prior papers to figure out what procedures might have been used in this one. I suggest providing full tables of the form of Nordlie et al, PLoS Computational Biol 2009.

*Response: We apologize for the lack of clarity. We have added extensive model details in the Methods section and the Appendix section, including basic model activation and learning functions, a summary of the general Leabra framework, and a full table depicting key model parameters used in this study. Note that “... equations and default parameters have been used to simulate over 40 different models in O’Reilly et al. (2012) and O’Reilly and Munakata (2000), and a number of other research models”, therefore, the current Theremin model was built on the architecture of Leabra and the previous hippocampal model ThetaPhase (Ketz et al., 2013). The detailed description of Theremin has been included in the Methods section, and we added implementation information in the Github repository provided so that replication of the paper results is more accessible.*

2. My second major thought is that to make small adaptations to a prior model in order to improve performance in a single task is not really newsworthy to any others than those who work on this somewhat niche sets of models. I think at least it should be shown that a second, more interesting phenomenon, such as the “testing effect” with partial information, which the authors say could arise from their model, should be validated. In this reviewer’s mind, that would make the paper stronger and a bit more general.

*Response: We appreciate the suggestion of adding a concrete model of a specific cognitive phenomenon consistent with the idea of error-driven learning in the hippocampus, and we strongly agree that by adding this simulation the paper could be stronger and more general. We have added two models of* ***testing effect*** *with and without the proposed error-driven learning mechanism in CA3, and showed that testing effect in the Theremin mimics testing effect in an empirical study that is also shown in the newly-added Figure 7, while testing effect in the NoEDL shows deficits in the testing condition. In accordance with our main idea that memory capacity and resistance to interference increase by incorporating the proposed biologically plausible error-driven learning mechanism in the hippocampal CA3, the testing effect also benefits from this mechanism in a way that when the list size is high (i.e., hitting the capacity limits), NoEDL model will not have the chance of utilizing error signals generated by comparing the retrieved pattern with the correct pattern to promote learning. On the other hand, the Theremin model will make use of the error signal to complete additional learning in the* ***retrieval practice*** *condition compared to the* ***restudy*** *condition (where less error is generated), therefore reaching a larger testing effect.*

*In general, we showed that this error-driven learning way of understanding the testing effect fits well with the behavioral understanding of the testing effect (Liu et al., 2021), and gets neural support as shown in the new simulations in this paper. We believe that this novel perspective will contribute hugely to the field of testing effect phenomenon, and promotes more exploration computationally and empirically on other learning phenomena in cognitive psychology and neuroscience.*

3. The equations shown are not mathematically rigorous. For example, equations 3 and 4 which indicate the rule used to update specific connection weights should indicate the two units (e.g. i and j) stating which is presynaptic, which is postsynaptic, and then how it depends on the firing rates r of either unit i or unit j.

*Response: Thank you for pointing that out. The original intention of equations 3 and 4 was to conceptually demonstrate the temporal-difference form of the error-driven learning used in the hippocampus model, with connections to the plus phase and minus phase used in the Boltzmann machine. We have modified the equations and main text related to those two equations in the paper accordingly.*

4. Equation 3 is particularly confusing as the difference in rates used is of vectors of different sets of units in entrorhinal cortex (layer 3 versus deep layers) which would not be the same size biologically speaking. Since it is impossible to take the difference of two vectors of different lengths, the authors must assuming identical numbers of units and then a one-to-one correspondence between units in deep layers with equivalent units in layer 3. Such important details and clear constraints/biological requirements for a model should be stated plainly so they can be tested.

*Response: We have clarified the nomenclature to show that it is actually the difference in activation state over a single population of neurons, ECout, when driven more strongly by different inputs (CA1 vs. ECin). In addition, we added relevant anatomical data about connectivity. In addition, we clarified the assumptions about the relationship between ECin and ECout in the model and the relevant anatomy.*

5. Since Equation 4 is the new one being used for this paper, again it would be better to see it written more accurately in terms of the time of firing rates. That is, is dW_ij for a connection from unit i to unit j equal to the firing rate of unit i in EC to the rate of unit j in CA3 at time t¬-tau subtracted from its rate at time t for a value of tau equal to a quarter of a theta cycle, so about 30ms? If this is the case (my best guess given what is presented) then it is essential that it is stated plainly and either evidence for such a plasticity rule provided, or a clear statement that there must be a process which responds to the *change* in firing rate over a 30ms period (rather than the absolute rate) so that ideally experimental groups could look for it and find the corresponding biochemical process, or those who model synaptic plasticity could suggest a mechanism for it, using known processes. Without such clear statements of the requirements for a model showing how it can be disproven, there is little benefit in adding alteration to alteration of a complicated model that may or may not correspond to the underlying biology. I see there are suggestions, perhaps based on work by Hasselmo’s group on different signs of plasticity at different phases in the theta cycle, but the exact requirements and equations are not provided.

*Response: The reviewer is correct that equation 4 (now equation 5) is at the heart of this paper and should be formulated and explained more thoroughly. We have modified the equation and related main text to more explicitly state that these are activity states of the CA3 neurons, driven by different inputs as a function of time. The extensive discussion thereafter has been revised in an attempt to more clearly elaborate the possibility that learning at this synapse is due to the temporal difference, and / or a form of heterosynaptic plasticity at this synapse, which has been demonstrated in at least some form.*

6. The text at the top of p.8 (which is far from Figure 1 on p.4, may need to be expanded and connected better with that figure’s caption.

*Response: We have elaborated that text.*

7. On p.9 the authors state they use in their default setting, a pretraining process that involved “turning DG and CA3 off”. While it is reasonable to assume there are cortical representations of words in our vocabulary, a bit more justification is needed, given its limited capacity, and the ease of plasticity and interference in hippocampal areas, to (1) state evidence for long-term hippocampal representations of vocabulary and (2) justification for essentially switching off hippocampal structures while such representations are acquired. Perhaps (2) is to ensure cortical representations arise (in EC) without (1) in HC, but if that is the case more explanation is needed, as it looks like EC to CA1 synapses are “trained” which would result in CA1 representations of long-term semantics.

*Response: If the hippocampus is to be capable of recalling information back into cortex, which is a core assumption of the Hebb-Marr framework (and most hippocampal theories), then it must be able to reactivate these cortical representations. That is the core assumption here. We have discussed this extensively in prior publications, and it is not new to this model. It is introduced earlier, in the paragraph starting: "We begin by briefly reviewing our earlier work showing how the monosynaptic pathway interconnecting the EC and CA1 can support error-driven learning,..." Given that this is not new to this model, we are not sure it would make sense to discuss more extensively here.*

8. The term “epoch” is not defined in the text or methods. Fig. 6 suggests 100 cycles per epoch? Is a cycle a theta cycle, about 100ms, so epochs are 10 sec long?

*Response: Sorry for the confusion, the clarification has been added into the Methods section – “Each pair was only trained once in each epoch, with each trial being a full theta cycle (~200 ms). All pairs (including AC pairs) were tested once in each epoch.” So the length of the epochs is dependent on the list size. For example, if the list size is 100, there will be 100 pairs of A-B associations that need to be trained, and 100 A-B + 100 A-C associations that need to be tested in one epoch, which will take longer than a list size of 20.*

9. p.16 “the difficulties are more at the level of abstract principles” – this statement is confusing, as it seems the models are so different at the biological level the “difficulties” are far from abstract. It is pretty easy to replace a plasticity rule in an architecture and test its consequences, or to find out what parameters are needed for the rule to work – nothing is abstract about that.

*Response: we agree this is a confusing statement, which does not add much meaning to the discussion. We removed it.*

10. The authors use in italics “ps” a lot when they I think mean p-values in significance tests? This is non-standard – I think just “p” is fine, but also state what sort of test is used.

*Response: The result report section has been modified according to the reviewer’s suggestions.*

# Reviewer #2:

(1)	The abstract and other places state the main problem with Hebbian learning as weight modification that continues "unnecessarily beyond" what is sufficient for retrieval. In some places, the authors refer to this naïve notion as the "simple Hebbian approach" (e.g., start of intro, para 4). While a simplistic ∆w=µxy rule encapsulates the old "fire together, wire together" dictum, I believe it is widely understood that such a learning rule is impractical, given its tendency to explode. That is, the foil in the authors' story is a straw man. The authors might have in mind some self-normalizing variant of generalized Hebbian plasticity (e.g., Oja's rule, BCM, etc.), but they don't mention it directly or discuss comparisons with other Hebb-like variations. The main issue with this is that implying "simple Hebb" to be an appropriate baseline for comparison is both unfair to Hebb and overgenerous to the authors' evaluation of their EDL models' performance. The paper's impact, and its connection to the comp/theory literature since the Ketz (2013) paper, would substantively benefit from clarifying throughout the manuscript precisely what is meant by "Hebbian learning", both in general discussion and when describing implementations in the 2013 model and this model (e.g., the ECin → DG pathway). Since the gap being studied here is Hebbian → EDL, any obscurity around "Hebbian" terminology detracts from the interpretability of the results.

*Response: Thank you for pointing out this very important question, which we have clarified in the revision. The reviewer is correct that without any self-limiting mechanisms the simple form of Hebbian learning will explode and it’s very unlikely to be the case in the brain. Therefore, when designing our previous model (Ketz et al., 2013), and carrying forward into this paper, we used the Conditional Principle Component Analysis (CPCA) Hebbian learning algorithm, which is based on the Oja variant for normalization and bounding.*

(2)	Relatedly, the scientific logic motivating the study of RW-style EDL rules established in the introduction (para 4) is rather weak. That logic could be paraphrased: "To avoid simple Hebb we need self-limited learning; 'one well-established class' is EDL; therefore we incorporate EDL and show that it outperforms simple Hebb." This logic undercuts itself at 'one well-established class', both because it's an appeal to authority and it hints at other classes which the authors fail to mention or study. This latter point raises questions (in a reasonable reader) of why this particular formulation of EDL was chosen for study from among the wider universe of self-limiting learning rules, whether well-established or not. Its relationship to error backpropagation is potentially interesting, but it doesn't substantiate its support in biology (which is fine, it's just that there are presumably many other self-limiting rules with varying levels of biological evidence and plausibility). If this paper is pushing us toward an "error-driven hippocampus", then what we (the field) need to see is the comparison between EDL rules and some spanning set of non-EDL self-limiting rules. (I might be mistaken, but I think the authors' NoEDL variant here simply disabled CA3 synaptic modification and the ThetaPhase variant utilized "Hebbian learning"; there is no test of non-EDL self-limited learning at those synapses.) If self-limited learning is the logical alternative to the Hebbian "mistake", then why isn't self-limited learning studied and explicated more broadly (within the context of this hippocampal architecture/model)? If EDL emerges as the self-limiting "winner" (on grounds of capacity, performance, or biological fidelity), then that makes a much stronger case. To fully support the authors' claims, I would suggest that they expand their investigation, to a reasonable extent, to provide some baseline of comparison with non-EDL self-limiting rules (e.g., FORCE learning, synaptic resource allocation, etc.).

*Response: As stated in our response to the previous comment, we did compare EDL vs. self-limited Hebbian learning rules used in our model. We also added this important further clarification in the discussion section: "As emphasized above, this interference is not a result of using a simplistic form of Hebbian learning without appropriate normalization and bounding --- the key failing of Hebbian learning that error-driven learning corrects is that it is purely local and autonomous and is not sensitive to an overall objective function that can determine when learning has accomplished its objective."*

(3)	The paper's title is perhaps too strongly worded, since "Hebbian Mistake" implicates Hebb in an error and conjures up something of a straw man (cf. comments 1&2 above). I can only suggest (sincerely suggest; I'm not demanding a title change here) that the authors reconsider whether the title strikes the right note given the study's claims.

*Response: Thanks for the comment and perspective. We added some discussion of the intention of the title: "Although we have provocatively characterized Hebbian learning as a mistake in order to highlight the error-correction nature of our alternative hypothesis, we nevertheless recognize that extensive research and modeling has productively leveraged the Hebbian principle to understand hippocampal function. Indeed, we have shown that the error-driven learning achieves much of the same overall learning objective, just through different means that improve learning performance and reduce interference."*

(4)	Figure 1: Please clarify the relationship between the 4 "quarters" and the theta cycle. It would be helpful to have a simple diagram showing the correspondence of Q1-Q4 aligned with a sinusoidal theta wave. Also, it looks like Q2 and Q3 are identical? Do those quarters correspond to the cycle 25-75 interval (with the big-loop signals) in Figure 6?

*Response: We revised the caption to explain this more clearly. Indeed, Q2 and 3 do not have differential pathway weighting, and do correspond to 25-75 cycles.*

(5)	Following on comment #3: It might be helpful to add a brief overview of the "nuts and bolts" of the model implementation (perhaps in the Methods section) that the authors could reference at this point, since many model details seem to be glossed over or presumed to be found in the 2013 paper. This should include at least brief descriptions of the units, activations, and layers an explanation of simulation timing relating time steps to cycles to quarters to epochs to train/test trials. These relationships won't be immediately obvious to most readers, and it shouldn't be necessary to go to the 2013 paper for basic information.

*Response: We have added extensive model details in the Methods section and the Appendix section, as well as a full table depicting non-default model parameters used in this study.*

(6)	p.5, following Eq (4): "ECin is _the_ sending activity into CA3"; I'm assuming that 'the' is a typo?

*Response: this was reworded along with the new clarified terminology.*

(7)	p.7, para 1: "Figure 1 shows the _standard_ hippocampal architecture...". The word 'standard' shouldn't really be used to describe these models. This architecture might be standard in the authors' labs, but there are many ways to construct models. There are other instances of "standard" in the manuscript which should be similarly struck.

*Response: We have modified the text about “standard” accordingly.*

(8)	While "NEpochs" and "ABmem" work fine as variable names, they are awkward to read and difficult to remember. The authors could just as well use "N" and "C" (or a similarly readable refactoring) to refer to training epochs and memory capacity.

*Response: The “NEpochs” and “ABmem” have been renamed to “N” and “M” for* ***N****umber of training epochs and* ***M****emory of AB after learning AC.*

(9)	Are the Theremin and ThetaPhase curves shown in Figure 4 the same as those in Figure 3? If so, that should be clarified, preferably in one or both captions. Or, the authors could reconsider the presentation of this data to prevent reader confusion.

*Response: They are the same curves shown for reference purpose in Figure 4. The clarification has been made in Figure 4 only, since it will probably make it very confusing to mention Figure 4 data in Figure 3.*

(10) Figure 5: The text does mention the difference in training epochs between Theremin (10) and NoEDL (30), but these plots with differently scaled x-axes in the left and right columns are not intuitively interpretable. Because the epochs are discrete, having fewer in the Theremin plots means that the visual slope of the connecting lines has a different meaning regarding the learning dynamics of the model w.r.t. the NoEDL plots. Given the relative complexity of the data (esp. the similarity curves), the ease of interpretation of this figure would greatly benefit from an attempt to horizontally stretch (half of) the plots so that the x-axis scales are preserved over all of them.

*Response: Thank you for the kind suggestion for increasing the readability of this paper. In fact, we originally plotted the Figure 5 in the exact same way the reviewer suggested, but abandoned the figure in our submitted version. The reason for that is, it is very hard (and not aesthetic) to plot the Theremin subplot (10 epochs) in a 30-epoch axis and see clearly what information it actually conveys. Also, the points being made here do not heavily require interpreting the slopes. To understand the similarity part of this figure, we compare the trend of Within AB and Within AC in the first half and the second half of learning across two models. Theremin is able to decrease the similarity due to powerful pattern separation ability endowed by the DG-driven EDL, but the NoEDL variant fails to do that and suffers from catastrophic interference. To understand the memory part of the figure, we focus on how much AB memory is left after successfully learning the AC. These points that the figure wants to convey does not require they are on the same x-axes, but does require more on the same y-axes. Again, we appreciate the kind suggestion, but we hope to keep our way of plotting the figure after consideration and discussion.*

(11) Figure 6: It would be very helpful to add some dotted lines or arrows to indicate the cycles at which DG input turns on (25). Also, something clearly happens around cycle 75 at the end of Q3, but the text and caption don't make this very clear. This could be clarified in the Methods as I suggested above or in other ways as the authors see fit.

*Response: Sorry for the confusion. The end of Q3, which is the start of Q4 (the plus phase), is when ECout patterns were clamped to ECin patterns for providing the ground truths. Figure 1 has been modified to better illustrate the temporal dynamics of the model, and details about model learning (including the meaning of Q4 plus phase) were added in the Sources of Error Driven Learning in the Hippocampal Circuit section.*

(12) Discussion, p.15, para 3 "Another class...": This paragraph discusses the long-running class of sequence learning models, but it doesn't come to a very satisfying conclusion. How exactly are we supposed to interpret the current study's results in the context of sequence learning, which depends precisely on the kinds of learned representational overlaps that the authors' CA3 EDL rule and EC → DG plasticity work to remove? The authors' seem to dismiss this entire literature by stating that "most" of these models synthesize predictions. If it's only most that can be thusly dismissed, then the ones that remain need to be addressed, no? How would Chip Levy's local context units fare in an EDL regime?

*Response: we agree that a more extensive and comprehensive discussion of these models in relation to EDL would be more satisfying. On the other hand, we don't think it makes sense for this paper to be the place for that, and yet we do think that we need to make some mention of this important literature in relation to our new model.*

(13) Discussion, Novel Predictions, second bullet point: Since the promise of experimental predictions was prominent in the abstract and other places, it's not clear why the authors did not already undertake to investigate some of these pathway-specific modulations in their model. As written, the predictions are very vague: "should affect error-driven learning", "the specific temporal dynamics associated...". Given the effort and expense of experimental corroboration, it would be more impactful to more clearly and precisely demonstrate hypothesis-driven results from pathway lesion/modulation interventions in the Theremin model to both inspire and guide experimentalists who may be interested in these questions.

*Response: Sorry for the confusion in our first draft. We have elaborated this second point in the Novel Prediction section. Specifically, what we meant is that “neural manipulations that selectively disrupt the theta cycle and / or these pathway-specific modulations should disrupt error-driven learning, therefore decreasing learning ability, but may not affect recall of previously-learned information to the same extent.” This is essentially what we have shown in this paper – the NoEDL variant assumes no such “pathway-specific modulations” and the learning performance is greatly compromised.*

# Reviewer #3:

1. One of the central goals of this model (and a previous model published by this group) is to adjust synaptic weights over the course of learning until EC­_OUT_ matches EC_IN_, with the specific addition of adjustments in this model until CA3 _DG_− CA3 _OUT_. I am familiar with the history of the CLS model and the advantage that the new adjustments in this model provide for reducing interference. The adjustments in the original version of this model (EC­_OUT_ matches EC_IN_) require a bit more explanation. What are the goals/advantages of having EC­_OUT_ match that of EC_IN_? This seems to imply that CA1 is being optimized to provide faithful representations of original EC input, rather than performing any unique transformations. The *dW* = CA1(EC­_OUT_ - EC_IN_) seems to negate somewhat the advantages afforded by *dW* = EC(CA3­_DG_ – CA3_NODGE_). In other words, the structure of *dW* = CA1(EC­_OUT_ - EC_IN_) seems like it disrupts the ability of CA1 to output unique separations, associations, or other transformations formed *within* the hippocampus. Moreover, it makes it challenging to incorporate strong recent evidence that entorhinal activity is heavily influenced by hippocampal activity. It might be interesting to probe CA1 activity patterns of EC _OUT_ without *dW* = CA1(EC­_OUT_ - EC_IN_) while still retaining *dW* = EC(CA3­_DG_ – CA3_NODGE_) to provide insight into how *dW* = CA1(EC­_OUT_ - EC_IN_) influences hippocampal computations/interference (and then change the name of the noEDL condition).

*Response: We hope that the updated terminology in equation 3 clarifies the confusion here, as also discussed in response to Reviewer #1's comments. We regret the confusion caused by the prior notation, which was unfortunate. The only requirement is that ECout retain a sufficient representation of ECin information to drive reactivation of corresponding cortical representations, and that ECin has some kind of reliable information-preserving projection into ECout.*

2. The authors attempt to leverage the unique timing of different inputs within a theta cycle in order to allow different patterns of activity to manifest across a region at distinct times. While I think it is a great idea to consider a major temporal organizing principle of the region, I am not sure the model needs to be nested within a theta timescale. Attempting to do so makes it fragile to scrutiny of the feasibility of the timescale and potentially less applicable to primate models where the theta rhythm is less prominent. The authors include some citations indicating that plasticity within a theta timescale may work (e.g. Hasselmo et al. 2002), but the work was targeting projections to CA1, which are not the target of their error-driven learning models. Synaptic plasticity of other synapses may obey very different temporal rules, and a more recent study they cite from Quirk et al. suggests that rhythms facilitating learning can be quite broad (i.e. <10Hz). Synaptic plasticity of other synapses may obey very different temporal rules. I think that the authors can maintain the theta cycle structure of the current model, but perhaps provide context that it is a valuable framework here for achieving their goals rather than *the* only hypothesized mechanism for error-driven learning. They can provide context in the introduction and discussion with any additional references justifying their timing and other possible timing mechanisms.

*Response: We appreciate this comment, and have also considered the potential for heterosynaptic plasticity mechanisms in addition to temporal dynamics, for driving the proposed form of synaptic plasticity. This was briefly mentioned before but we have elaborated and clarified this in the revision.*

3. The authors do a great job explaining the relevant connectivity. However, there are occasions where it is not clear which connections are undergoing Hebbian learning and which connections are undergoing error-driven learning. For example, there are places in the text where the language can read as if there is EDL at CA3 inputs to CA1. Figure 1 is extremely helpful in detailing the connections that are giving rise to expected patterns of activity at different phases of the theta cycle. Perhaps this figure can be adapted to indicate (perhaps through dotted or dashed lines versus solid lines) which connections have EDL, which ones have Hebbian learning, and which (if any) have no plasticity. In addition, perhaps patterns of activity can be labeled as CA3_DG_ (or “target”) versus CA3_NODGE_ (or “expected”) when they occur in the upper panels, and similarly for other patterns utilized for EDL. Adding the equations next to the connections undergoing EDL would also be tremendously helpful.

*Response: The suggested separations of EDL and Hebbian learning, as well as no learning projections, have been made in Figure 1 by using solid, dashed, and dotted lines. As much as we would like to provide extra useful information in the Figure (e.g., label such as “target”), it is hard to depict both CA3 and CA1 EDL in this way, because CA1 minus phases for different projections are at different quarters. Therefore, we chose to explicitly write that in the caption, specifically specifying plus and minus phases for each of the EDL projections.*

4. The acronym Theremin, while fun, is an odd combination of letters from the model name, has an extra E, and perhaps doesn’t capture the description or goals of the model well. I understand that model names are often somewhat personal, but I think that a different name would increase the impact and accessibility long-term. In addition, as there are only two synapses with EDL, I suggest removing the word Total from the model name.

*Response: Thank you for the suggestion -- after careful discussion, we still think we will stick with the name Theremin (forgiving the extra E). One clarification to be made here, also in our response to your Major Comment 3, is that there are more than two synapses with EDL. To be more precise, the previous model (Ketz et al., 2013) made CA3->CA1, ECin->CA1, and CA1<->ECout EDL, shown in equation 6 and equation 7 in that paper. The current paper made two more synapses (ECin->CA3, CA3->CA3) EDL, shown in the modified Figure 1.*

5. I find it hard to interpret the tables at the end of the manuscript, particularly the testing table. I am not exactly sure what is to be learned from these table. If no input is given for the B/C pool, then why do several pools have Context B#/C# labels? In addition, B#/C# goes to 4, when this notation is used in the test to indicate a single pair of a # of possible pairs much greater than 4. Perhaps the two tables can be combined into a figure detailing how the inputs to the a subset or all pools evolve across training and test.

*Response: Sorry for the confusion. The Context B#/C# pools are list context for helping the model pattern separate two lists to achieve better resistance to interference. The “#” was simply used to differentiate those four context pools, and either “B#” or “C#” would be chosen for a single trial depending on the type of list being tested. To better illustrate this point, we have included an example figure of training and testing patterns with labels. These figures, together with the tables, should be enough for readers to understand the experimental settings.*

6. The authors experiment with plasticity at EC-DG synapses, and their results suggest that favoring LTD increases separation/reduces interference. Perhaps the authors could relate this finding to what is known about at these synapses (Hsu et al., *The dentate gyrus as a filter or gate: a look back and a look ahead*, 2007)

*Response: We appreciate the suggestion that helps the interpretations of the current findings. We have addressed the point in the Discussion section.*

7. The authors relate there model to several other models and comment on the ability of the hippocampus to generate predictions. It would be interesting if they could also relate their model to Jung et al. 2018 (*Remembering rewarding futures: A simulation‐selection model of the hippocampus*).

*Response: The Jung et al. 2018 paper is a thorough review of how hippocampus might be performing a simulation-selection process, and offers a theoretical model supporting their claims. The related part is, from the authors’ understanding, whether the hippocampus generates novel activity patterns for prediction of future events. The Theremin model suggests that computationally, from the standard CLS perspective, the hippocampus is not suitable for generating predictions, which would require abstracting long-term regularities from multiple experiences. Instead, it does fast learning, and therefore supports episodic memory. The Jung et al. 2018 paper was built on work suggesting that the hippocampus could represent never-experienced trajectories during rest/sleep states (e.g., Gupta et al., 2010), as well as other theoretical models that we have discussed in the Discussion section. While this is a fascinating possibility, we still believe that given the biological constraints (e.g., # of neurons in the hippocampus) and the computational constraints (e.g., the nature of the hippocampal learning system), it is very unlikely that the hippocampus would support prediction per se, instead it could be highly involved in any decision making and prediction making processes, given its role of episodic memory storage. We have discussed the relevance of the current paper to Jung et al., 2018 in the Discussion section.*

5. There is a typo in the Figure 2A description “traind.”

*Response: Thank you for pointing that out. The typo has been fixed.*
