# Supplementary material for: Correcting the hebbian mistake: Toward a fully error-driven hippocampus
Source: PLoS Comput Biol. 2022 Oct 11;18(10):e1010589. doi: 10.1371/journal.pcbi.1010589 (PMC9586412; doi:10.1371/journal.pcbi.1010589)
Supplement: Attachment — Submitted filename: responses_sub3.docx [file pcbi.1010589.s008.docx]

Thank you for the positive response to our prior revisions. Here we describe our revisions to the second round of reviews.

# Editor:

**In addition, it seems that Reviewer # 2's concerns about implementing additional forms of self-limiting learning rules to compare with the performance of error-driven learning was not fully addressed. Because of this, the claims of the manuscript seem too strong in some places and could be toned down.**

*We attempted in the revision to make it clear that our prior Hebbian implementation that we compare against implements "industry standard" forms of bounding (Oja and BCM). However, to address this further concern, we revised the emphasis of the distinction between Hebbian and error-driven in terms of the more specific issue of learning driven specifically by overall memory success, rather than the more general framing that could have been confused with these various forms of bounding.*

# Reviewer #1:

# 1. A couple of minor suggestions: Eq.2: When using the sum notation it is standard to include the subscripts (W_ij, x_j then sum over j) otherwise it could simply be written as a matrix multiplying a vector without an explicit summation.

*We are trying to keep the math as simple as possible (there are studies about including too much math in the main part of a paper being a bad thing :), and just trying to convey the essential relationships. If we add subscripts here, we presumably need to add them everywhere for consistency, which tends to just add clutter -- these are not implementational equations -- just conceptual. If we adopt a particular form of matrix notation, then we need to introduce that, and again it is not really the point. If the editors feel strongly about this, we can certainly do it, but anyway that was our reasoning.*

*2.* P.4 "how testing effect" I think "how the testing effect"

*fixed.*

3. Fig 7 caption "gained undesirable memory performance in RP compared to RS" : I think you mean the opposite. At least RS has high performance compared to RP in the right-hand columns. Maybe you mean it "lost desirable memory performance" which has a slightly different meaning as what is "desired" is to match the data. i.e. there is no gain in performance in RP.

*Reworded.*

4. Fig.8: I think it needs a bit more labeling in the figure, which pools are items etc, and more in the caption why some blocks are silent during the "test" , which blocks represent A or B or C etc

*Labels added.*

# Reviewer #3:

# 1. For example, the authors write "building on theta-phase dynamics discovered by Hasselmo, Bodelon, and Wyble (2002)," but do not elaborate on the specific theta-phase dynamics they will be leveraging (although readers can probably figure this out by pulling up the citation and reading the paper).

# *We added an explanation.*

# In another example, the authors write "Second, this error-driven learning could arise from heterosynaptic plasticity in the hippocampus (Lee, 2022)." The authors should state what potential heterosynaptic mechanisms exist (e.g. evidence of heterosynaptic plasticity by which neuromodulators at different phases of the theta cycle) and provide citations. The paper they cite is a model comparing successor representation learning algorithms to the "heterosynaptic plasticity rule." I think it would improve readability for the authors to go through the manuscript and determine if there are places where a citation is provided instead of important details + citations.

*We explained the meaning of our use and have the detailed references immediately following. The Lee reference was removed.*
